# Impact of COVID-19 pandemic on greenhouse gas and criteria air pollutant emissions from the San Pedro Bay Ports and future policy implications

**DOI:** 10.1088/1748-9326/ad7747

**Published:** 2024-10-07

**Authors:** Jiachen Zhang, Junhyeong Park, Nancy Bui, Sara Forestieri, Elizabeth Mazmanian, Yucheng He, Cory Parmer, David C Quiros

**Affiliations:** 1 Department of Civil and Environmental Engineering, University of Southern California, Los Angeles, CA 90089, United States of America; 2 California Air Resources Board, 1001 I St #2828, Sacramento, CA 95814, United States of America

**Keywords:** vessel congestion at ports, surge in cargo throughput, Ports of Los Angeles and Long Beach (San Pedro Bay Ports), COVID-19 pandemic, clean transportation policy, ground-based freight transportation, Air pollutant emissions inventory

## Abstract

The Ports of Los Angeles and Long Beach, collectively known as the San Pedro Bay Ports, serve as vital gateways for freight movement in the United States. The COVID-19 pandemic and other influencing factors disrupted freight movement and led to unprecedented cargo surge, vessel congestion, and increased air pollution and greenhouse gas emissions from seaport and connected freight system operations beginning in June 2020. In this study, we conducted the first comprehensive monthly assessment of the excess particulate matter, oxides of nitrogen (NO_x_), and carbon dioxide (CO_2_) emissions due to the heightened congestion and freight transport activity from ocean-going vessels (OGVs), trucks, locomotives, and cargo handling equipment (CHE) supporting seaport operations. Excess emissions peaked in October 2021 at 23 tons of NO_x_ per day and 2001 tons of CO_2_ per day. The strategic queuing system implemented in November 2021 significantly reduced the number of anchored and loitering OGVs and their emissions near the ports, even during continued high cargo throughput until Summer 2022. Looking forward, we analyzed projected emissions benefits of adopted California Air Resources Board regulations requiring cleaner and zero-emission trucks, locomotives, and CHE over the next decade. If a repeated port congestion event were to occur in 2035, NO_x_ emissions from land-based freight transport should be lessened by more than 80%. Our study underscores the potential emissions impacts of disruptions to the freight transport network and the critical need to continue reducing its emissions in California and beyond.

## 1. Introduction

The Ports of Los Angeles (LA) and Long Beach (LB), also known as the San Pedro Bay Ports, serve as vital gateways for freight movement and handle around 30% of all containerized trade via seaports in the United States (Port of Los Angeles 2023). While the Ports of LA/LB are crucial for supplying consumer goods and bolstering the economy, seaport and connected freight system emissions worsen regional air quality and disproportionately impact disadvantaged communities in the South Coast Air Basin (SCAB), which is one of the non-attainment areas for the National Ambient Air Quality Standards for ozone and particulate matter (PM) in the United States (US EPA 2024).

Port-related activities, encompassing the operation of ocean-going vessels (OGVs), cargo handling equipment (CHE), trucks, and locomotives, are a significant source of air pollution including nitrogen oxides (NO_x_), PM (Mousavi *et al* 2018), and volatile organic compounds (VOCs). These pollutants and ozone (produced from NO_x_ and VOCs) can lead to respiratory diseases (e.g. asthma and bronchitis) and cardiovascular diseases (Zhao *et al* 2017, Zhang *et al* 2019). Low-income, often minority-populated communities are more likely to be located near seaports, rail yards, or freeways and are therefore at a greater risk of exposure to air pollution from freight transport (Rowangould 2013), leading to environmental health disparities (Houston *et al* 2008, Douglas *et al* 2019, Meng *et al* 2021, Lane *et al* 2022). Particularly, four disadvantaged communities that are also designated as AB 617 communities in the SCAB are significantly impacted by freight movement and the operation of the Ports of LA/LB (SCAQMD 2022). Port-related activities are also a significant source of CO_2_ emissions, contributing to global climate change (Alamoush *et al* 2022).

Given the significance of the Ports of LA/LB and their emissions, it is critical to effectively mitigate seaport emissions and their negative air quality and climate impacts while ensuring that they continue to provide economic benefits and deliver essential goods for California and much of the United States. The Ports of LA/LB developed a Clean Air Action Plan in 2006, and released a Clean Air Action Plan update in 2017 (Port of Los Angeles, Port of Long Beach n.d.). This plan, along with other regulations adopted by the California Air Resources Board (CARB) and policies implemented by the International Maritime Organization (Xiao *et al* 2022), have significantly reduced emissions from port operations (US EPA 2021). The emissions inventories for the Ports of LA/LB indicate a 64% reduction in NO_x_ and a 12% reduction in CO_2_ from 2005 to 2020, despite a 23% increase in cargo throughput (Port of Los Angeles 2021).

The COVID-19 pandemic in 2020, shifts in consumer behavior, record-breaking import volumes, and labor shortages disrupted the supply chain and normal flow of freight movement in the Ports of LA/LB (Deeb and Leonardo 2023). Although decreases in transportation emissions induced by COVID-19 restrictions in 2020 reduced nitrogen dioxide (NO_2_) and ozone concentrations in the SCAB (Schroeder *et al* 2022), supply chain disruptions led to a backlog of ships waiting to unload in 2021 at the Ports of LA/LB, resulting in increases in NO_2_ and PM_2.5_ concentrations near the ports (Skipper *et al* 2024).

A critical question that arises is: how can the Ports of LA/LB enhance their capacity to manage sudden surges in cargo, increase their resilience to unforeseen events, and prevent sudden increase in emissions if an unexpected congestion happens in the future? To address this, we conducted a thorough evaluation of the emission impacts of port congestion and various mitigation strategies.

This study provides the first comprehensive monthly assessment of the excess PM, NO_x_, and CO_2_ emissions during the seaport (hereafter, also referenced as ‘port’) congestion period, as compared to a business-as-usual scenario, covering not only OGVs but also trucks, locomotives, and CHE. Our research also investigates the impact of various strategies to mitigate emissions associated with increased port activities. Specifically, we assessed the effectiveness of the queueing system implemented by the Pacific Maritime Management Services (PacMMS) in reducing emissions from OGVs near the coast. Moreover, we developed a scenario to project emission increases in a hypothetical port congestion event in 2035 to evaluate the effectiveness of implementing CARB’s adopted mobile source regulations that will further control tailpipe emissions and greenhouse gas emissions. Our findings provide insights into strategies for mitigating potential future significant emission spikes during unforeseen surges in cargo volumes at ports.

## 2. Methods

### 2.1. Study scope

**Figure 1.**
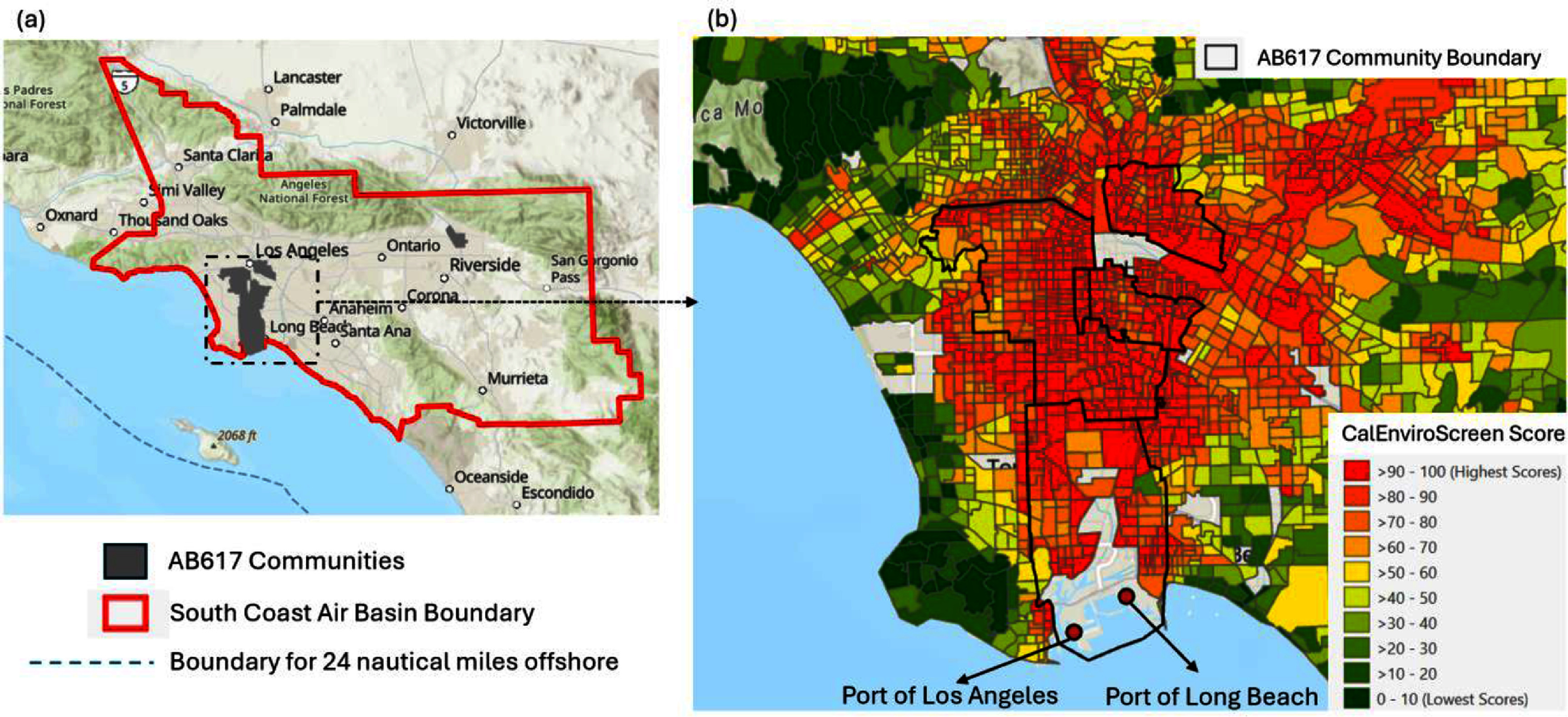
(a) The South Coast Air Basin, our study area for analyzing emissions from land-based sources, is delineated by the red line. The blue dashed line represents 24 nautical miles offshore, which encloses our study area for analyzing emissions from ocean-going vessels. (b) A zoomed-in map displaying the Ports of LA/LB and AB 617 designated environmental justice communities near the goods movement corridors in Southern California, overlaid with the CalEnviroScreen 4.0 (CES4.0) Cumulative Impact (CI) score (OEHHA 2023). Higher percentiles of the CES4.0CI score in California indicate greater environmental justice burden by considering environmental pollution burden and population vulnerability characteristics.

This study estimates emissions from vessel activities within 24 nautical miles off the coast of the SCAB, emissions of CHE that operate at the Ports of LA/LB, and emissions of locomotives and trucks that frequently visit the Ports of LA/LB in the SCAB (figure 1). Figure 1 also shows the close proximity of AB 617 designated environmental justice communities to freight corridors in Southern California, which underscores the need for reducing port-related emissions to safeguard community health.

We quantified the emissions during the port congestion period from 2020–2022 (hereafter referred to as CONGESTION emissions) using observed activity data that reflects the increased cargo throughput and port congestion at the Ports of LA/LB. To assess excess emissions during the port congestion period, we also developed a business as usual (BAU) scenario for the 2020–2022 period, which serves as a counterfactual scenario assuming the unexpected port congestion had not occurred. This scenario uses 2019 as the base year for projection and takes into account economic growth in the forecast of activities for trucks and locomotives. For OGVs, BAU is defined as emissions during the first half of calendar year 2020. The difference of CONGESTION minus BAU is calculated as the excess emissions due to the port congestion and increased freight throughput.

### 2.2. Methodology for developing emission inventories

We assessed air pollutant emissions from port-related activities of the following four source categories:

(1) OGVs, operating within 24 nautical miles (nm) from the Southern California shoreline. We report OGV emissions from container vessels, tankers, and cruise ships, which account for the majority (∼90%) of vessel emissions. Emissions from other vessel types, including tugboats, represent less than 10% of OGV emissions and were not significantly impacted by the port congestion event. Note that CO_2_ emissions beyond the 24 nm boundary could also contribute to global climate change, but we focused on assessing the excess emissions arising from the prolonged waiting time near the shore.
(2) Locomotives, including Class I line haul locomotives and switchers
(3) CHE, including 14 types of equipment (table S1 in supplementary material), such as rubber-tired gantry cranes, container handling equipment such as top picks and side picks, and yard trucks at the Ports of LA/LB
(4) Drayage trucks that visited the Ports of LA/LB

In a simplified form, emissions of pollutant *i* for each source category are calculated as

\begin{equation*}{\text{E}_i} = \mathop \sum \limits_{{j}} {\text{E}}{{\text{F}}_{{{i}},{\text{j}}}}{ } \times {\text{ Activit}}{{\text{y}}_{{j}}}\end{equation*}

where EF*_i_*_,*j*_ is the emission factor of pollutant *i* (i.e. NO_x_, PM, or CO_2_) when a source category (i.e. OGVs, locomotives, CHE, or trucks) is operating in *j* mode/process, and Activity*_j_* quantifies the operation of the source category in *j* mode/process. For example, the emission factor of trucks during the running exhaust mode is in the unit of g/mile and activity of the running exhaust is in the unit of miles. For OGVs, we accounted for excess emissions when vessels were in different modes (i.e. at berth, at anchorage, and loitering). Note that the actual calculations were more detailed than equation (1), because within each source category, we applied different emission factors for different engine model years and also considered different types of CHE and locomotives. For example, for the NO_x_ emissions of port trucks, we took the sum of emissions of trucks of different model years during the start exhaust, running exhaust, and extended idling processes. Section 2.3 provides data sources and basis for the emission factors and activities of different source categories for different scenarios.

### 2.3. Datasets used for emissions inventory development

Data sources for activity and emission factors for the BAU scenario are shown in table 1. Emission factors for NO_x_, PM, and CO_2_ from locomotives and CHE were obtained from the corresponding sectors of CARB’s models for off-road mobile sources (California Air Resources Board 2021a, 2022a, 2022b). Emission factors for OGVs were obtained from U.S. EPA (US EPA 2020). Emissions for trucks visiting the Ports were obtained from CARB’s EMFAC2021 model (California Air Resources Board 2021b); we used data for the ‘POLA Class 8’ category from EMFAC output, which represents heavy-duty trucks that frequently visit the Ports of LA/LB. EMFAC and the sector-specific off-road models (California Air Resources Board n.d.) used in this study are the official emission inventory models for California’s on-road and off-road mobile sources, respectively. These emission inventory models are widely used by academics, public agencies, and industry, as well as serving as the scientific foundation of CARB’s policy designs and recommendations, playing a pivotal role in shaping California regulations that impact various industries. Note that while the emissions inventories were developed based on the best available datasets, there are inherent uncertainties in the emission factors and activity data. For example, testing for emission factors is very expensive and typically can only be done for a limited number of trucks, which are then used to parameterize and model emission factors across different truck model years. Similarly, the idle time of drayage trucks were estimated based on the best available data from loggers, which only covers a small subset of the drayage fleet (California Air Resources Board 2021b).

**Table 1.** Data sources for activity and emission factors of different source categories.

Source category	Data source for CONGESTION Activity	Assumptions for BAU Activity	Data source for emission factors
Ocean-going vessels	Automatic Identification System (AIS) from National Oceanic and Atmospheric Administration (NOAA)	AIS data for the January-May period of 2020	U.S. Environmental Protection Agency’s 2020 updates to OGV emission factors
Cargo handling equipment (CHE)	Twenty-foot Equivalent Unit of containers (TEU) data from the Ports of LA/LB	Projection of CARB’s CHE emissions inventory	CARB’s CHE emissions inventory
Locomotives	TEU data from the Ports of LA/LB	Projection of CARB’s Locomotive emissions inventory	CARB’s Class I linehaul locomotive emission inventory and Air Emission Inventory from Ports of LA/LB24
Trucks	Data of trucks visiting the ports from the Ports of LA/LB	CARB’s EMFAC model projection based on the TEU growth rate estimated by the Ports of LA/LB	CARB’s EMFAC2021 model

Emissions for CONGESTION were calculated by scaling up BAU emissions by the ratio of observed CONGESTION activity to BAU activity for each source category. Emission factors for each process of each source category were assumed to be the same between the CONGESTION and BAU scenarios, so the only difference between these two scenarios was their different activities. Excess emissions resulting from port congestion and increased freight throughput were calculated by subtracting the BAU emissions from the CONGESTION emissions.

Table 1 shows the data sources for estimating the CONGESTION activities for different sectors. The activity for OGVs was based on data from the Automatic Identification System (AIS) published by National Oceanic and Atmospheric Administration (NOAA 2022a, 2022b, 2023). AIS provided minute-by-minute speed and coordinate locations of each vessel, which were matched with vessel registry information to estimate emissions from all engines, generators, and boilers on-board each vessel. Increases in the activities of CHE and locomotives were estimated based on the cargo throughput data from the Ports of LA/LB. The Ports of LA/LB also provided data for the number and frequency of trucks visiting the Ports.

## 3. Results

### 3.1. Increased cargo throughput and vessel activity

**Figure 2.**
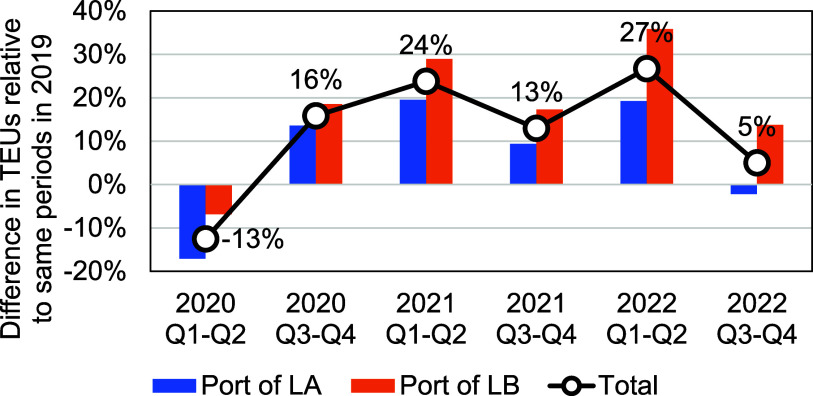
Percentage increase in cargo throughput (in TEUs) at the Port of LA, Port of LB, and the total cargo throughput of the Ports of LA/LB during 2020–2022, compared to the corresponding periods in 2019. The percentages provided in text represent the change in total cargo throughput for the Ports of LA/LB.

Twenty-foot Equivalent Unit of containers (TEU) volume is a standardized maritime industry measurement used when counting cargo containers of varying lengths. We obtained TEU data from the Ports of LA/LB and compared TEU volume for the full years beginning in 2020 through the end of 2022 with the same period in 2019 (figure 2). The TEU volume in the first and second quarters of 2020 was 13% lower than in 2019, while the TEU volume in the second half of 2020 through the end of 2022 was higher than the same periods in 2019. The average monthly TEU volume during the cargo surge period (July 2020–June 2022) was approximately 20% higher than the average in 2019.

**Figure 3.**
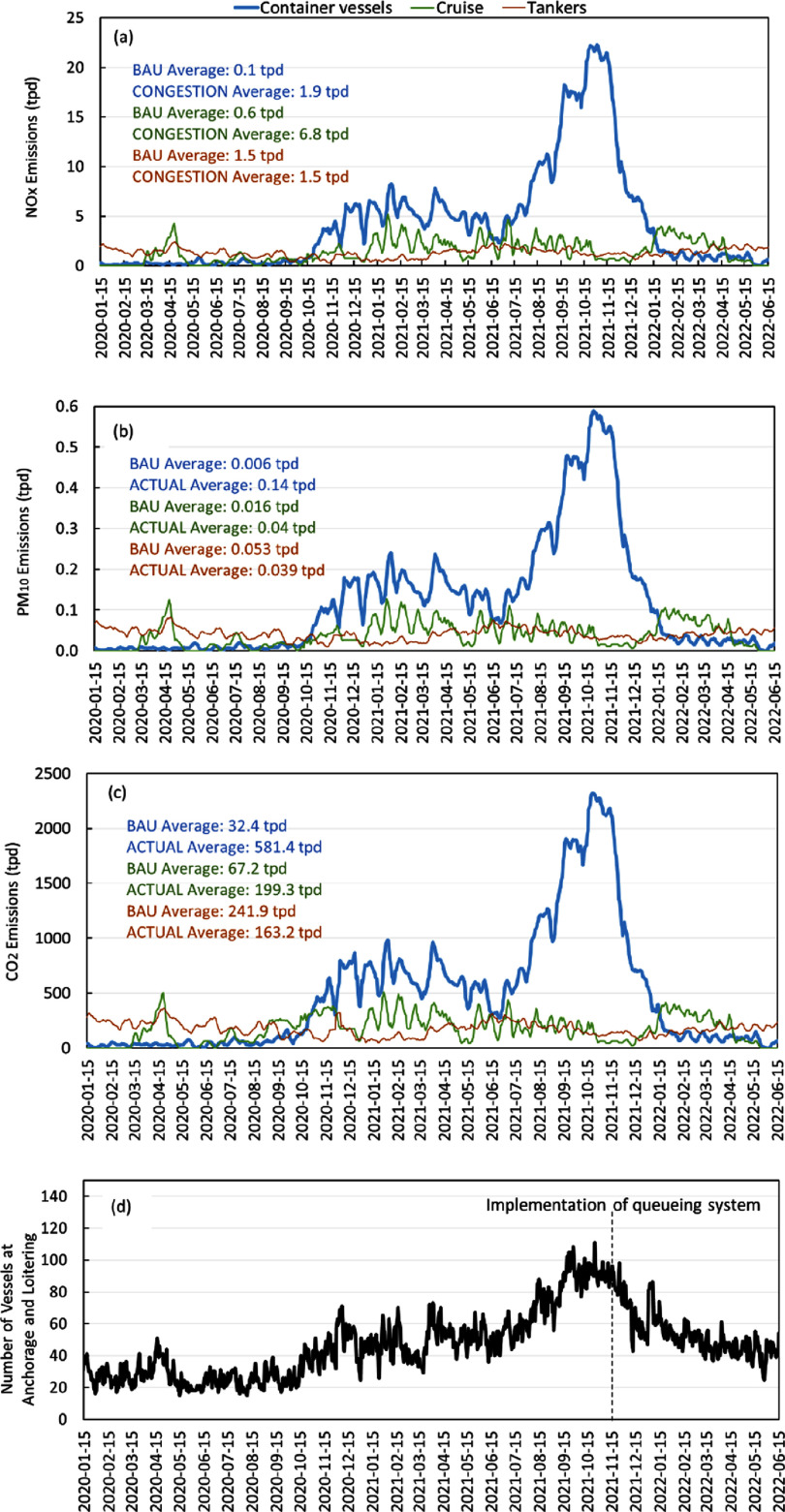
Daily CONGESTION emissions (tons per day, or tpd) of (a) NO_x_, (b) PM_10_, and (c) CO_2_ from at-berth, anchored, and loitering container vessels, cruise vessels, and tankers at the Ports of LA/LB. Seven-day moving average emissions based on AIS observations (three days prior, the day of, and three days after) are presented. Average BAU and CONGESTION emissions for each vessel category during the port congestion period (July 2020–June 2022) are shown in text. (d) shows the daily number of vessels at anchorage and loitering within 24 nautical miles off the coast.

The surge in freight movement and resulting congestion led to an abnormally increased number of vessels waiting for berths at the Ports of LA/LB. By November 2021, there was a historically high number of container vessels at anchorage and loitering (figure 3(d)). In response, on 16 November 2021, PacMMS implemented a queuing system to encourage vessels to wait for a berthing assignment outside of the ‘Safety and Air Quality Area’, which is 50 or 150 nautical miles off the shore of California (depending on direction of travel into the vicinity of the ports). The queuing system calls for vessels to voluntarily drift beyond 50–150 nm offshore when they do not have a berthing assignment within 72 h. Additionally, it places vessels on the queue list when they depart from their last port of call, allowing them to transit at slower speeds rather than crowding in congested waters near the Ports of LA/LB while waiting for a berthing assignment (Pacific Maritime Management Services 2022). After the implementation of the queueing system in November 2021, loitering of container vessels dissipated and the number of vessels at anchorages and loitering trended toward pre-pandemic levels (figure 3(d)), despite that freight movement was still at record-high levels of 1.7 million TEUs per month in the first two quarters of 2022, 27% higher than in the same period in 2019 (figure 2).

### 3.2. Excess emissions from vessels due to port congestion

Figure 3 shows the CONGESTION and BAU emissions of container vessels, cruise vessels, and tankers while they were at-berth, anchored, and loitering near the shore. Emissions from the continuous operation of auxiliary engines and boilers used by the record number of OGVs significantly increased in late 2020 through early 2022. Specifically, the at-berth, anchored, and loitering activities of container vessels generated NO_x_ emissions of 5.1 tons per day (tpd) averaged over the port congestion period, while their BAU emissions were only 0.1 tpd. PM_10_ and CO_2_ emissions were a factor of 22 and 18 higher than BAU emissions, respectively. Anchorage and loitering activities for cruise and tanker vessels were not as significantly influenced by the port congestion as container vessels. The anchorage and loitering activities of cruise vessels and associated emissions of CONGESTION were on average about a factor of three higher than BAU. Tanker vessels did not experience significant variability in anchorage and loitering activities since 2020, and their emissions remained near BAU average levels.

Notably, since the implementation of the new queuing system in November 2021, the number of anchored and loitering vessels significantly decreased (section 3.1). The implementation of the queuing system significantly reduced vessel emissions near the coast (figure 3). Both our study and Vukić and Lai (2022) estimated that emissions from vessels at anchorage reached their peak sometime around late October to early November 2021. Although emissions from queueing vessels were redirected to another geographic region, which is not a complete mitigation strategy for excess emissions, especially greenhouse gas emissions, we highlight two air quality benefits of the queueing system. First, by directing emissions further offshore, greater dispersion could occur prior to transport to populated land areas. Second, as discussed in section 3.1, enlisting on the berthing assignment queue after vessels departed their last port of call could have enabled slower transiting, which provides fuel savings and greenhouse gas emissions reductions.

### 3.3. Excess emissions from land-based freight transport

**Figure 4.**
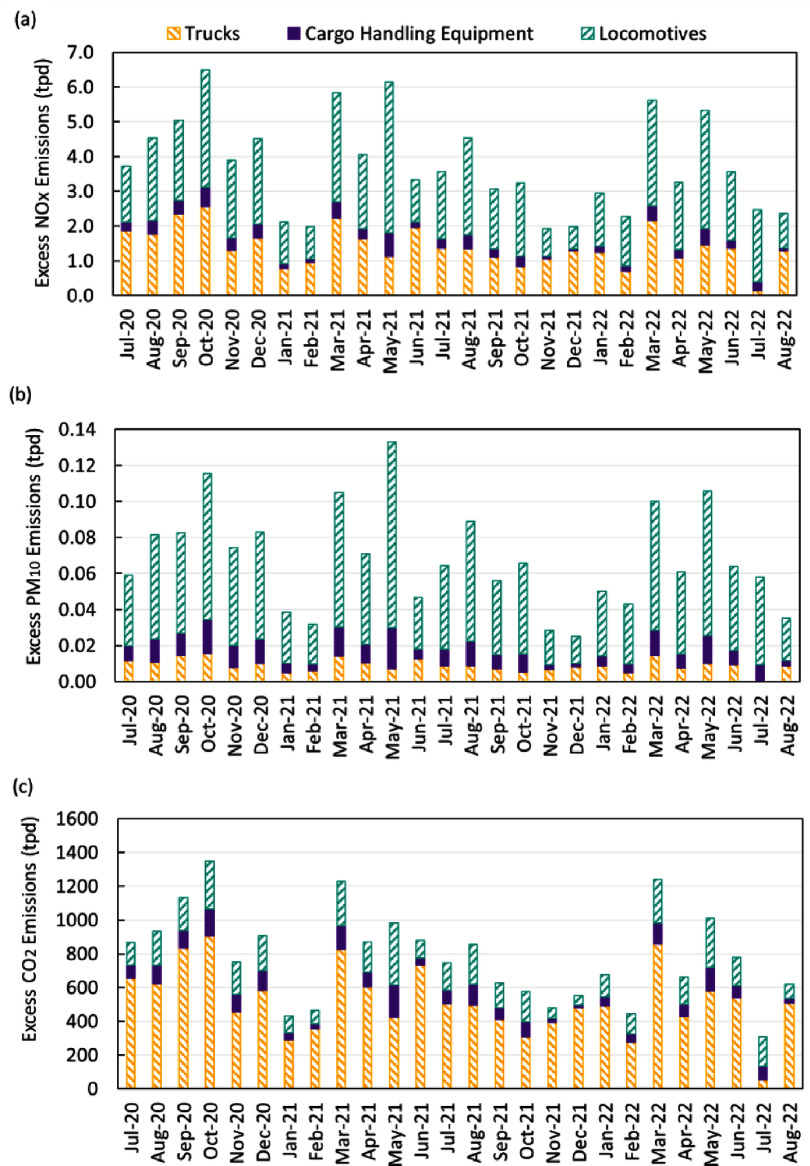
Monthly excess tailpipe emissions (CONGESTION minus BAU) of (a) NO_x_, (b) PM_10_, and (c) CO_2_ from ground freight movement by trucks and locomotives visiting the Ports of LA/LB, as well as cargo handling equipment at the Ports of LA/LB. Unit of emissions is tons per day (tpd).

In addition to OGVs, land-based freight transport (i.e. trucks, locomotives, and CHE) also produced higher emissions than the BAU scenario due to the surge in the demand of cargo transportation. Figure 4 shows their ‘excess emissions’ (i.e. the additional emissions due to the port congestion event relative to BAU operations). Locomotives were the largest source of excess NO_x_ and PM emissions, while trucks were the main contributor to excess CO_2_ emissions, suggesting that the emission factors of locomotives on per kg fuel basis are higher than trucks.

## 4. Discussion and policy implications

**Figure 5.**
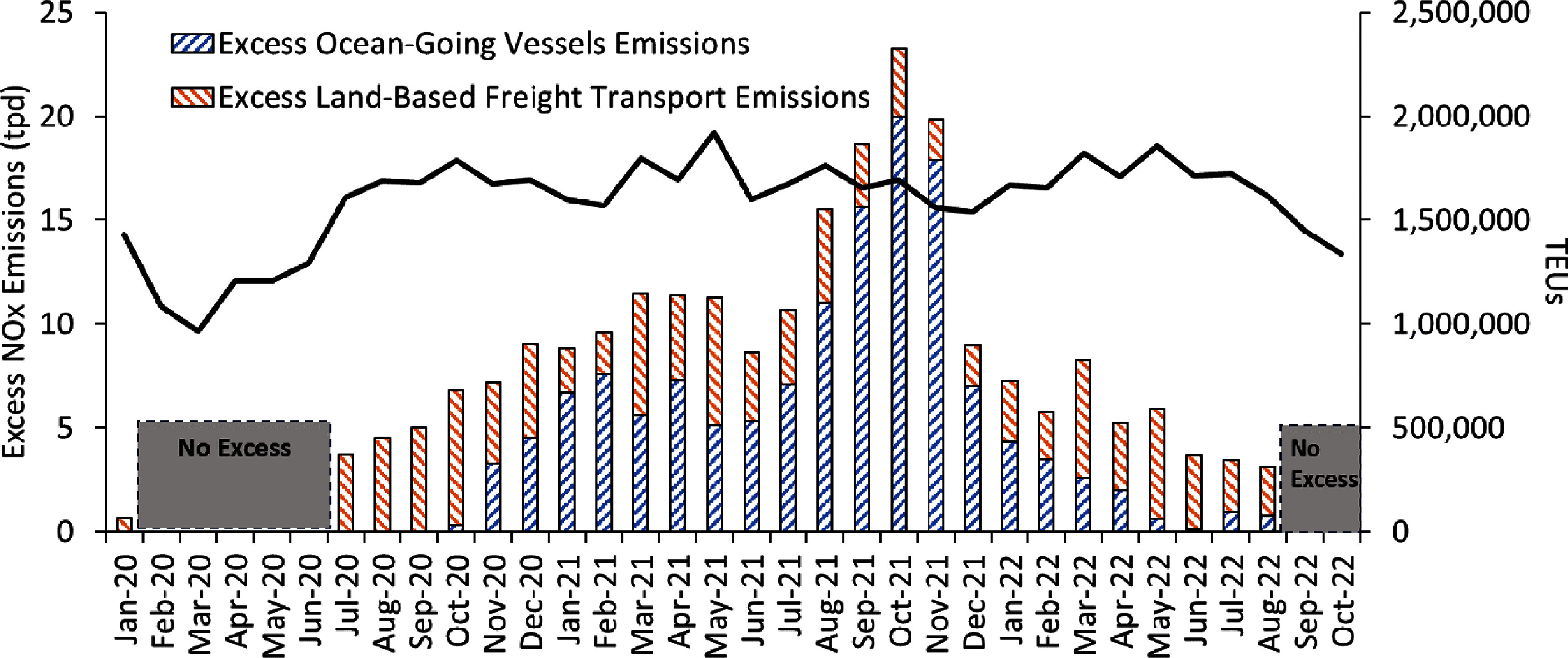
Monthly total number of TEUs being moved through the Ports of LA/LB and excess NO_x_ emissions (in the unit of tons per day, tpd) of OGVs and land-based freight transport (including trucks, cargo-handling equipment, and locomotives) from 2020 to 2022.

We found that the Ports of LA/LB experienced an unprecedented surge in cargo from late 2020 to early 2022, leading to significantly increased emissions of air pollutants and GHGs at and surrounding the Ports of LA/LB. Combining all these port-related sources, total NO_x_ emissions peaked at 23 tons per day (tpd) in October 2021 (figure 5). To put this number into perspective, total NO_x_ emissions from all sources (including stationary, area, and mobile sources) in the SCAB were estimated to be 350 tpd in 2018. The carrying capacity of NO_x_ is 60 tpd in 2037, which means that NO_x_ emissions need to be controlled under 60 tpd to attain the federal 70 parts per billion (ppb) 8 hr ozone standard (South Coast Air Quality Management District 2022). Hence, repeated future occurrences of port congestion events could jeopardize attainment of federal ozone standard standards.

The Ports of LA/LB also found that GHG emissions from all seaport-related activities had been steadily declining since 2005 because of continuous port, state, and international efforts to curb emissions; however, greenhouse gas emissions in 2021 surpassed the records in 2005 (Port of Los Angeles 2022). The heightened emissions during this unexpected seaport congestion event highlights the importance of increasing the ability of California’s freight transport system to accommodate surges in cargo without resulting in as significant increases in emissions.

For OGVs, the queueing system implemented by PacMMS was proven to be an effective way of reducing excess emissions near shore. As of this publication, PacMMS has kept the queuing system in place, but could discontinue the program at their discretion. Unless the queuing system is discontinued, to the extent a similar congestion event occurs in the future, the ports may be better prepared to minimize excess emissions from OGVs.

For land-based freight transportation, CARB has historically and recently adopted regulations to reduce the emissions of trucks, locomotives, and CHE. To explore the influence of these regulations, we estimated the excess emissions for a hypothetical CLEAN scenario, assuming the same proportional increase in cargo throughput as CONGESTION, but with the full implementation of adopted CARB regulations and projected baseline activity growth in 2035. Regulations being considered in the CLEAN scenario include the recently adopted Advanced Clean Fleets regulation that requires all drayage trucks to transition to zero-emission by 2035 and the In-Use Locomotives Regulation that promotes cleaner locomotives technology. The CLEAN scenario also reflects the benefits of the current CHE regulation that has been fully implemented since 2017, plus additional natural turnover to Tier 4 Final equipment by 2035. This what-if scenario analysis quantifies the potential reduction in excess emissions from land-based freight transport due to more stringent emission reduction regulations. The Supplementary Materials include more detailed methodology for projecting emissions for the CLEAN scenario.

**Figure 6.**
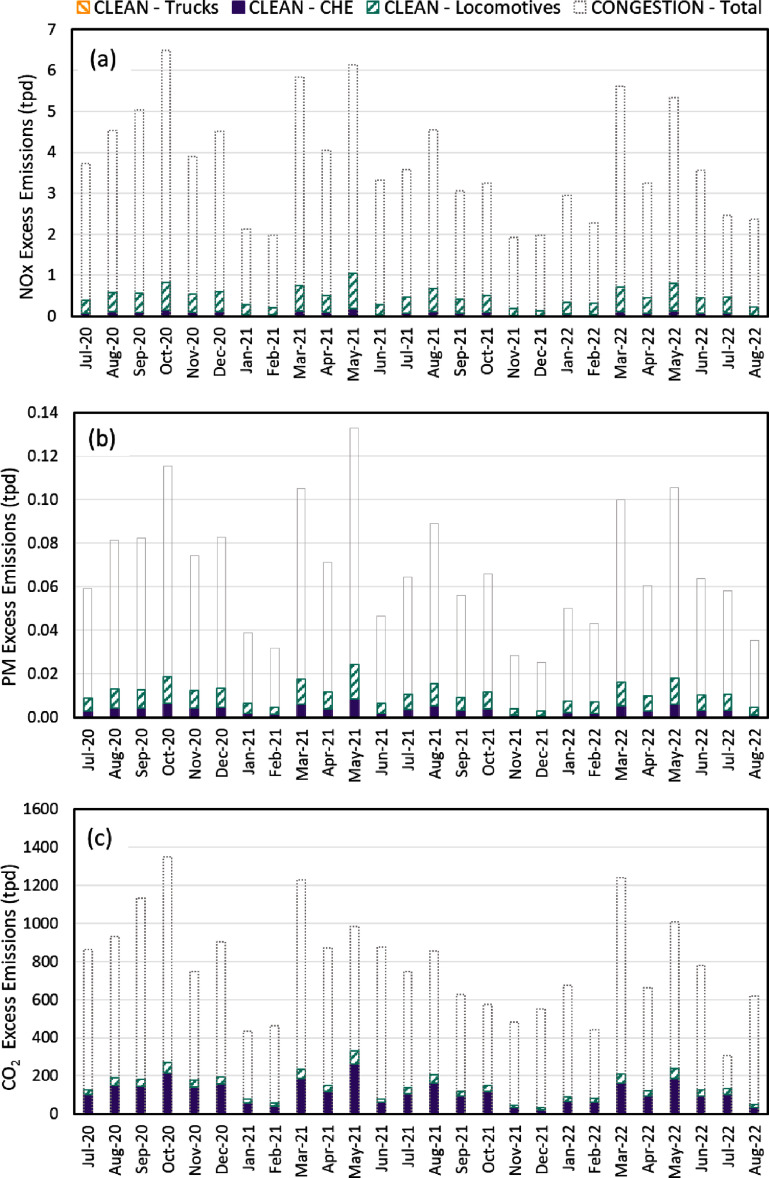
Projected excess tailpipe emissions (tons per day, or tpd) of (a) NO_x_, (b) PM_10_, and (c) CO_2_ for trucks, cargo handling equipment (CHE), and locomotives for the CLEAN scenario, in comparison to the total excess emissions of land-based sources for the CONGESTION period (represented by bars with dashed gray lines) that occurred in 2020–2022.

As shown in figure 6, excess NO_x_, PM, and CO_2_ tailpipe emissions due to the potential future surge in cargo throughput would decrease significantly by 87%, 84% and 81% from 2022 to 2035, respectively. Trucks visiting the ports will have zero tailpipe emissions in 2035, because the Advanced Clean Fleets regulation requires all drayage trucks transition to a zero-emission powertrain by 2035. Note that we also estimated excess non-tailpipe emissions for CONGESTION and CLEAN scenarios in figure S1 in supplemental material. For CHE, natural turnover to more engines meeting Tier 4 Final standards will lower NO_x_ and PM emission factors for the fleet at large, but because no CO_2_ emission standards were included, CO_2_ emissions are not expected to significantly decline based on currently adopted regulations. Excess NO_x_ and PM emissions from CHE would be much lower in 2035 than 2022 due to the turnover to Tier 4 Final equipment. Conversely, the CO_2_ emissions in 2035 would be higher than those in 2022, due to the projected port activity increase from 2022 to 2035. Overall, the excess emissions would be significantly lower in 2035 than 2022. Note that there are emissions associated with fuel production and manufacturing cleaner trucks, locomotives, and equipment. Assessing their emissions is important but is beyond the scope of this study, which focuses on the tailpipe emissions from mobile freight sources in the SCAB.

Note that CARB’s In-Use Locomotive regulation and the Advanced Clean Fleets regulation (included in the CLEAN scenario) require waivers from the U.S. Environmental Protection Agency (EPA) to be fully enforced. Without federal initiatives to curb emissions in off-road mobile sources, obtaining waivers and authorizations from the U.S. EPA is crucial for CARB to effectively regulate and mitigate freight-related emissions. As per the Clean Air Act, other states in the U.S. have the option to adopt CARB’s mobile source regulations, which are more stringent than federal regulations. For states where port-related activities are a major source of emissions, we recommend they also adopt CARB’s regulations.

## 5. Conclusions

The freight transport of containerized goods through the Ports of LA and LB, also known as San Pedro Bay Ports, decreased in early 2020 relative to pre-pandemic levels. However, the trend reversed by the end of 2020, with increased freight movement continuing into 2021, reaching a historically high number of OGVs at anchorage and loitering near the coast in November 2021. This study innovatively combined California’s official emission inventories with the up-to-date activity data to assess the impact of this unforeseen port congestion and conducted scenario analyses to evaluate the effects of clean transportation policies in the event of future cargo surges.

This study for the first time provides a monthly assessment of the excess emissions originating from different mobile sources including OGVs, trucks, locomotives, and CHE at and surrounding the Ports of LA/LB. We found that vessel congestion and increased freight volumes at the Ports of LA/LB resulted in remarkable increases in emissions, with excess NO_x_ emissions of all sources peaking at 23 tons per day in October 2021. The implementation of a queuing system in November 2021 was found to significantly reduce the number of loitering and anchored vessels as well as vessel emissions near the coast.

Further, we investigated future potential of freight system disruptions on air quality in 2035 after the implementation of the recently adopted In-Use Locomotive and Advanced Clean Fleets regulations. Our results show substantially reduced emissions from freight transport through the Ports of LA/LB should a similar port congestion happen in the future. This study highlights the importance of continued regulation of the emissions of all components of the freight system. We recommend other states and countries with major ports adopt similar regulations as those being adopted in California. In addition to providing emissions reductions and public health protections to communities adjacent to major freight transport corridors during typical operations, continued regulation, implementation, and enforcement of air quality regulations provide additional protections during future potential congestion or other supply chain disruption events.

## Data Availability

All data that support the findings of this study are included within the article (and any supplementary files).

## References

[erlad7747bib1] Alamoush A S, Ölçer A I, Ballini F (2022). Ports’ role in shipping decarbonisation: a common port incentive scheme for shipping greenhouse gas emissions reduction. Cleaner Logist. Supply Chain.

[erlad7747bib2] California Air Resources Board (2021a). 2021 line-haul locomotive emission inventory. https://ww2.arb.ca.gov/sites/default/files/2022-07/2021%2520Line-Haul%2520Locomotive%2520Emission%2520Inventory%2520%2528Final%2529%25202022%2520July%2520Update.pdf.

[erlad7747bib3] California Air Resources Board (2021b). EMFAC2021 technical document.

[erlad7747bib4] California Air Resources Board (2022a). 2022 cargo handling equipment emissions inventory.

[erlad7747bib5] California Air Resources Board (2022b). 2022 Class I Switcher Rail Yard Emission Inventory. https://ww2.arb.ca.gov/sites/default/files/2022-07/2022%2520Class%2520I%2520Switcher%2520Emission%2520Inventory%2520technical%2520document%252007112022.pdf.

[erlad7747bib6] California Air Resources Board (n.d.). OFFROAD emissions model. https://arb.ca.gov/emfac/offroad/emissions-inventory/b3ec728421af9bb0eeaa6f40e1988ee702fb2ec4.

[erlad7747bib7] Deeb N, Leonardo A (2023). Exploring the impact of Covid 19 on the maritime transport sector. IOP Conf. Ser.: Earth Environ. Sci..

[erlad7747bib8] Douglas J A, Archer R S, Alexander S E (2019). Ecological determinants of respiratory health: examining associations between asthma emergency department visits, diesel particulate matter, and public parks and open space in Los Angeles, California. Preventive Med. Rep..

[erlad7747bib9] Houston D, Krudysz M, Winer A (2008). Diesel truck traffic in low-income and minority communities adjacent to ports: environmental justice implications of near-roadway land use conflicts. Transp. Res. Rec..

[erlad7747bib10] Lane H M, Morello-Frosch R, Marshall J D, Apte J S (2022). Historical redlining is associated with present-day air pollution disparities in U.S. Cities. Environ. Sci. Technol. Lett..

[erlad7747bib11] Meng Y-Y, Su J G, Chen X, Molitor J, Yue D, Jerrett M (2021). Improvements in air quality and health outcomes among california medicaid enrollees due to goods movement actions. Res. Rep..

[erlad7747bib12] Mousavi A, Sowlat M H, Hasheminassab S, Pikelnaya O, Polidori A, Ban-Weiss G, Sioutas C (2018). Impact of particulate matter (PM) emissions from ships, locomotives, and freeways in the communities near the ports of Los Angeles (POLA) and Long Beach (POLB) on the air quality in the Los Angeles county. Atmos. Environ..

[erlad7747bib13] NOAA (2022a). AIS data for 2020. https://coast.noaa.gov/htdata/CMSP/AISDataHandler/2020/index.html.

[erlad7747bib14] NOAA (2022b). AIS data for 2021. https://coast.noaa.gov/htdata/CMSP/AISDataHandler/2021/index.html.

[erlad7747bib15] NOAA (2023). AIS data for 2022. https://coast.noaa.gov/htdata/CMSP/AISDataHandler/2022/index.html.

[erlad7747bib16] OEHHA (2023). CalEnviroScreen 4.0. https://oehha.ca.gov/calenviroscreen/report/calenviroscreen-40.

[erlad7747bib17] Pacific Maritime Management Services (2022). Container vessel queuing process FAQs for the ports of Los Angeles, Long Beach, and Oakland. https://pacmms.storage.googleapis.com/wp-content/uploads/2023/04/21191335/Container-Vessel-Queuing-Process-FAQs-for-LA-LB-OAK.pdf.

[erlad7747bib18] Port of Los Angeles, Port of Long Beach (n.d.). 2017 clean air action plan update. Clean Air Action Plan.

[erlad7747bib19] Port of Los Angeles (2021). 2020 air emissions inventory highlights. https://kentico.portoflosangeles.org/getmedia/32625bc2-958b-40f8-8c1e-27ff624d6406/2020_Air_Emissions_Inventory_Highlights.

[erlad7747bib20] Port of Los Angeles (2022). 2021 air emissions inventory highlights. https://kentico.portoflosangeles.org/getmedia/d100eedb-a492-4109-8102-0fe2aa57b19c/2021_Air_Emissions_Inventory_Highlights.

[erlad7747bib21] Port of Los Angeles (2023). Facts and Figures|Statistics|Port of Los Angeles.

[erlad7747bib22] Rowangould G M (2013). A census of the US near-roadway population: public health and environmental justice considerations. Transp. Res. D.

[erlad7747bib23] SCAQMD (2022). Chapter 8: environmental Justitice. 2022 Air Quality Management Plan.

[erlad7747bib24] Schroeder J R, Cai C, Xu J, Ridley D, Lu J, Bui N, Yan F, Avise J (2022). Changing ozone sensitivity in the South Coast Air Basin during the COVID-19 period. Atmos. Chem. Phys..

[erlad7747bib25] Skipper N T, Kaiser J, Odman M T, Hasheminassab S, Russell A G (2024). Local scale air quality impacts in the Los Angeles Basin from increased port activity during 2021 supply chain disruptions. Environ. Sci.: Atmos..

[erlad7747bib26] South Coast Air Quality Management District (2022). 2022 air quality management plan. https://www.aqmd.gov/docs/default-source/clean-air-plans/air-quality-management-plans/2022-air-quality-management-plan/final-2022-aqmp/final-2022-aqmp.pdf?sfvrsn=16.

[erlad7747bib27] US EPA, U (2024). Current nonattainment counties for all criteria pollutants. https://www3.epa.gov/airquality/greenbook/ancl.html.

[erlad7747bib28] US EPA (2020). Ports emissions inventory guidance: methodologies for estimating port-related and goods movement mobile source emissions. https://nepis.epa.gov/Exe/ZyPDF.cgi?Dockey=P10102U0.pdf.

[erlad7747bib29] US EPA (2021). Case study of the san pedro bay ports’ clean air action plan 2006–2018: best practices and lessons learned. https://www.epa.gov/sites/default/files/2021-03/documents/420r21011.pdf.

[erlad7747bib30] Vukić L, Lai K (2022). Acute port congestion and emissions exceedances as an impact of COVID-19 outcome: the case of San Pedro Bay ports. J. Shipping Trade.

[erlad7747bib31] Xiao G, Wang T, Chen X, Zhou L (2022). Evaluation of ship pollutant emissions in the ports of Los Angeles and long beach. J. Mar. Sci. Eng..

[erlad7747bib32] Zhang J, Wei Y, Fang Z (2019). Ozone pollution: a major health hazard worldwide. Front. Immunol..

[erlad7747bib33] Zhao L, Liang H-R, Chen F-Y, Chen Z, Guan W-J, Li J-H (2017). Association between air pollution and cardiovascular mortality in China: a systematic review and meta-analysis. Oncotarget.

